# A Study on Pattern of Retinal Detachment in Patients with Choroidal Coloboma and Its Outcome after Surgery at a Tertiary Eye Hospital in Nepal

**DOI:** 10.1155/2019/7390852

**Published:** 2019-04-21

**Authors:** Barsha Suwal, Govinda Paudyal, Raba Thapa, Sanyam Bajimaya, Sanjita Sharma, Eli Pradhan

**Affiliations:** Vitreo Retina Service, Tilganga Institute of Ophthalmology, Kathmandu, Nepal

## Abstract

**Background:**

To review the pattern of retinal detachment (RD) in patients with choroidal coloboma and type of reattachment surgery performed and to study its outcome in terms of retinal reattachment, visual acuity, and postsurgical complications.

**Methods:**

Observational case series of a single tertiary eye institution of 13 eyes having choroidal coloboma with RD done from January 2015 to June 2017.

**Results:**

Mean age of presentation was 29.3 years (Range 14–60 years). Males were two times more affected than females (2.25 : 1). The overall rate of anatomic success achieved after RD repair and silicon oil removal at 6 months was 92.3% (12/13 eyes). Following surgery, visual acuity improved in 6 out of 11 eyes (54.54%), remained unchanged in 4 eyes (36.36%), and worsened in 1 eye (9.1%). The most common complication following surgery was secondary glaucoma in 30.7% (4/11 eyes).

**Conclusion:**

The overall anatomic success rate of retina reattachment surgery in colobomatous eye is good, and the visual outcome following surgery can improve in majority of the cases or may remain same in few cases. Hence, timely surgery is advocated. But careful follow-up is required as the risk of postoperative complications is also high.

## 1. Background

Ocular coloboma is a rare ocular malformation which occurs due to defective closure of the embryonic fissure, which normally occurs in the sixth and seventh week of gestation [1]. Coloboma may involve the iris, ciliary body, lens, choroid, retina, and optic nerve. A coloboma of the choroid is characterized by congenital absence of part of the retinal pigment epithelium and choroid.

Patients with posterior segment colobomas are at risk of rhegmatogenous retinal detachment (RRD) occurring as a consequence of retinal breaks located: (a) outside the area of the coloboma, (b) inside the anomalous retinal tissue within the coloboma, or (c) both [2]. Various studies have reported that RRD occurs in 2.4–42% of patients that have choroidal coloboma [3–9]. This can occur in the abnormally thin colobomatous retina or extra colobomatous area. Although spontaneous retinal reattachment has been reported in the literature, most retinal detachments associated with chorioretinal coloboma require surgery and often have poor visual outcomes [4, 7].

Herein, we retrospectively review the pattern of retinal detachment in patients with choroidal coloboma and type of reattachment surgery performed and study its outcome in terms of retinal reattachment, visual acuity, and postsurgical complications.

## 2. Research Design and Methodology

It was an observational case series study of a single tertiary eye center done from January 2015 to June 2017. Patients above the age of 10 years with retinal detachment related to choroidal coloboma were included in the study. Patients with other retinal problems were excluded from the study. Detailed history and examination were done as per the pro forma. Choroidal coloboma was classified according to Ida Mann Classification, which is stated as follows [10]:

  Type 1: coloboma extending above the anatomic disc
  Type 2: coloboma extending up to the superior border of disc
  Type 3: coloboma extending below the lower border of disc
  Type 4: coloboma involving the disc only
  Type 5: coloboma present below the disc with normal retina above and below the coloboma
  Type 6: pigmentation present in the periphery
  Type 7: coloboma involving only the periphery

Type of retinal reattachment surgery performed was noted, along with the presence of retinal break. Follow-up on first postoperative day, one week, one month, and 6 months was done. At each follow-up, visual acuity and status of the retina were recorded. Postoperative complication, if any, was also noted. Ethical clearance was obtained from the Institutional Review Board of Tilganga Institute of Ophthalmology, and it also conformed to the provisions of the Declaration of Helsinki 1995.

## 3. Results

A total of 13 eyes of 12 patients were included in the study. Mean age of presentation was 29.3 years (range 14–60 years). Males were two times more affected than females (2.25 : 1). Iridochoroidal coloboma was bilateral in 11 eyes. RD was present in 7 right eyes, 4 left eyes, and bilateral in 1 patient. History of parental consanguinity was absent in all the patients, while only one patient confirmed history of prophylactic laser therapy. There was no history of previous RD in same or fellow eye. Associated ocular anomaly was present in all the eyes, most common being iris coloboma (11 eyes), cataract (6 eyes), microcornea (4 eyes), nystagmus (3 eyes), and strabismus (3 eyes). Type 3 coloboma (53.84%) was the most common type followed by type 1 (38.46%).  Table 1 summarizes the patient characteristics.

Table 2 shows the surgical record of the patients. The surgical approach was 23-gauge pars plana vitrectomy with silicon oil tamponade (1000 centistokes) with Endolaser photocoagulation around the margin of the coloboma in all the cases, around the break where a retinal break is visible, and all around the vitreous base (360°) when a break is not visible. None of the patients had scleral buckling procedure. Bullous RD was present in all the cases. Retinal break was identified preoperatively or intraoperatively in 69% of the cases. Six eyes with RD (66.66%) had retinal break within the intercalary area, two eyes had retinal break in the retinal periphery (22.22%), and one eye with RD (11.11%) had retinal break both within the intercalary area and in the retinal periphery. Posterior vitreous detachment was induced during surgery by suction using vitreous cutter or silicone tipped cannula over the edges of the optic nerve head in all the cases. Perfluorocarbon was used to stabilize the retina during surgery which was removed at the end of surgery. Lensectomy was done in 6 eyes (46.15%) which had existing cataract that affected the visibility of the posterior segment while undergoing surgery and to aid in better trimming of the vitreous base in eyes with proliferative vitreoretinopathy (PVR). Membrane peeling was also done in 3 cases (23.07%) with PVR. Patients were advised for face-down position following surgery for 3 weeks. We lost 2 patients on follow-up after 6 months. The oil was ultimately removed in all the remaining eyes after 6 months, and one eye had concurrent secondary scleral fixation intraocular lens at 6 months follow-up while undergoing silicon oil removal.

Anatomic success in this series was defined as the retina remaining attached at the last follow-up visit. The overall rate of anatomic success achieved after RD repair was 92.3% (12/13 eyes).

**Figure 1:**
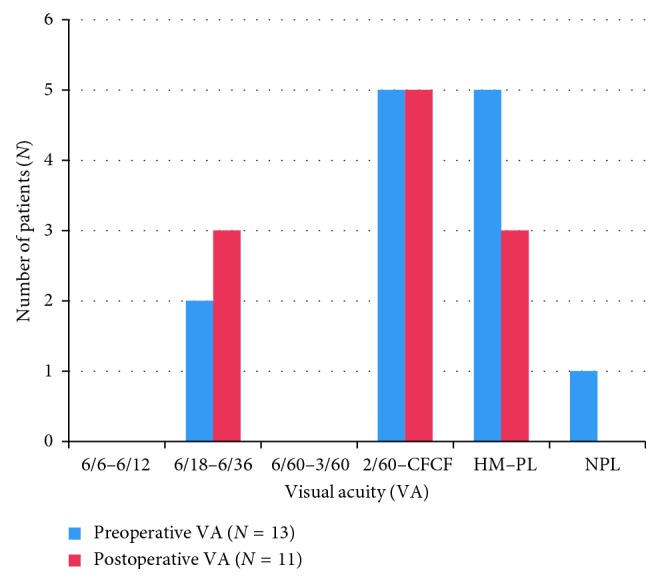
Comparison of visual acuity pre- and postoperatively. Abbreviations: *N*: number; VA: visual acuity; CFCF: counting finger close to face; HM: hand movement; PL: perception of light; NPL: no perception of light.

**Figure 2:**
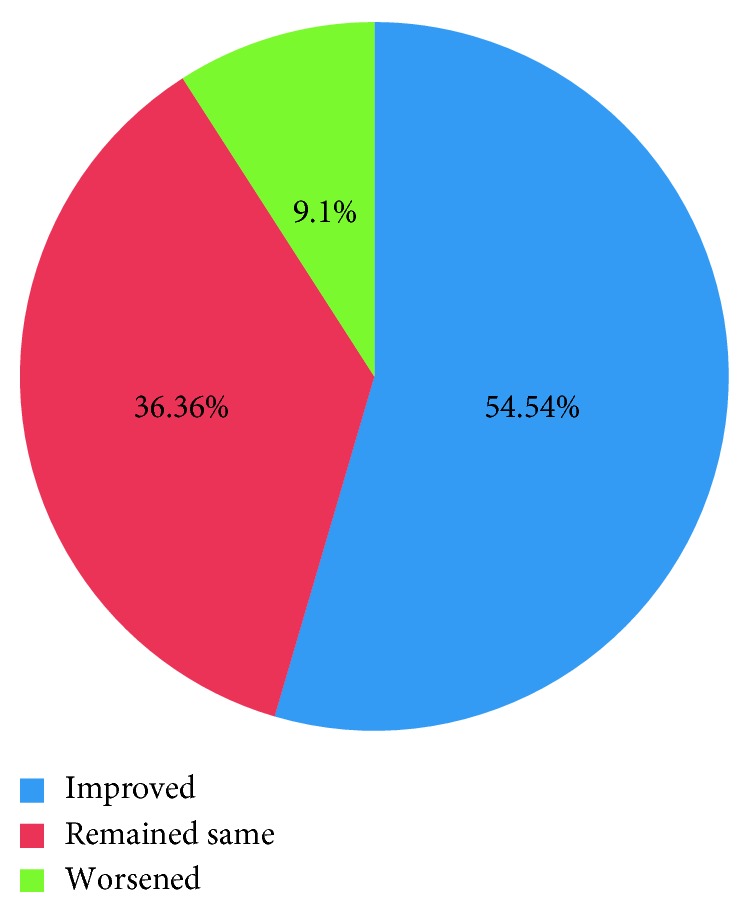
Outcome of visual acuity (VA) after surgery.

Figure 1 summarizes the VA before and after surgery, and Figure 2 summarizes the improvement in VA outcome after surgery.

63.64% of the eyes had complications in the postoperative period. Of the complications, four eyes (30.7%) had secondary glaucoma, one eye (7.69%) had recurrent retinal detachment, one eye (7.69%) had cataract, and one eye (7.69%) had band-shaped keratopathy.

Table 3 summarizes the postsurgical complications.

## 4. Discussion

Our study evaluated the pattern of retinal detachment in patients with choroidal coloboma. Also the primary outcomes (anatomical success and visual recovery) and secondary outcome (complications) in these patients were measured.

### 4.1. Clinical Demographics

In the series described by Mann, the most frequent coloboma extended to and beyond the superior aspect of the optic nerve and likely involved the papillomacular fibres [10]. In our study, coloboma extending below the lower border of disc (type 3) was the commonest type, followed by coloboma extending above the anatomic disc (type 1).

Retinal detachment attributable to coloboma requires a central break in the inner and outer layer at the margin of the coloboma. Retinal breaks were identified preoperatively and intraoperatively in 9 out of 13 eyes (69%) in our study. The most common site for break and detachment has been reported to be the neurosensory retina within the coloboma (referred to as intercalary membrane) [4, 6]. In our study, 6 eyes had break in intercalary membrane and 1 eye had break both in the intercalary membrane and in the normal retina. So, a total of 7 out of 9 eyes (77.79%) had break in the ICM, which could be related pathologically to coloboma. Two eyes had retinal break outside the detached retina in the retinal periphery (22.22%). Presence of retinal break in the retinal periphery suggests abnormal vitreoretinal adhesion away from the coloboma. Since, in 30.76% of the eyes, retinal break could not be identified, we suggest that prophylactic barrage laser should be given both around the margin of the coloboma and around the vitreous base which is also supported by other studies [3].

The breaks that cause retinal detachments in colobomatous eyes are often difficult to localize because of poor contrast due to absence of retinal pigment epithelium and choroid and associated nystagmus. Other factors include presence of atrophic holes without the presence of flaps or operculae in the thin rudimentary retina and hidden breaks in overhanging edge of coloboma or in areas of haemorrhage [11, 12]. Additionally, in colobomatous involvement of the optic disc, fluid may enter the subretinal space through defects in the optic nerve tissue. Identifying breaks is challenging but vital for optimum management. A high degree of suspicion and meticulous examination under a wide-angle viewing system is required.

### 4.2. Primary Outcomes

In our study, anatomic success after RD repair was 92.3% at the last follow-up visit (1 week after silicon oil removal at 6 months) which is comparable to other reports which had a success rate that ranging from 35 to 100% at the last follow-up visit [3, 7, 11–21].

Most of the eyes (45.45%) had a final best-corrected visual acuity (BCVA) CF CF-2/60. However, 27.27% of the patients' VA improved to 6/18–6/36, and 27.27% had HM-PL. Choroidal coloboma involved the macula and the optic disc in 6 out of 13 eyes (46.15%) each, which could explain the poor visual prognosis in these patients. Also, presence of coexistent strabismus, nystagmus, microcornea, and cataract could be accounted for poor visual outcome. Even though there was guarded visual prognosis following surgery, we found that following surgery, VA actually improved in 6 out of 11 eyes (54.54%), remained unchanged in 4 eyes (36.36%), and worsened in 1 eye (9.1%). A previous study on pediatric RD showed that VA improved in 56.7% of the eyes following surgical repair [22]. Likewise, another review of pediatric RDs found that 38% of eyes that underwent surgical repair had equal or better BCVA compared to their fellow eye at the final follow-up [23]. Hence, surgical repair is advocated in these patients.

### 4.3. Secondary Outcome

63.64% of the eyes had postoperative complications. Among them, the commonest was secondary glaucoma (30.7%), followed by recurrent retinal detachment, cataract, and band-shaped keratopathy (7.69% each). Other articles also cite secondary rise in intraocular pressure to be the commonest complication following surgery which can range from 11.9% to 48% [13, 20, 21]. In our study, all glaucoma patients were managed with topical antiglaucoma medicines. The cause of redetachment in one eye was a missed break in retinal periphery along with PVR changes. It was managed with silicon oil removal, membrane peeling, laser application around the break and edge of the coloboma, and silicon oil (5000 centistokes) reinjection. Following silicon oil removal after 6 months, the eye maintained a BCVA of 2/60 and attached retina.

Subsequent silicon oil removal may not be possible in up to 70% of the cases [3, 12, 21]. Hence, management of retinal detachment in the setting of coloboma is challenging and often complex [4]. Difficulties we encountered in managing these cases included localization of retinal breaks and creating adequate chorioretinal adhesion.

The limitations of the study were its retrospective nature, small number of patients, and short follow-up duration.

## 5. Conclusion

The overall anatomic success rate of retina reattachment surgery in colobomatous eyes is good, and the visual outcome following surgery can improve in majority of the cases or may remain same in few cases. Hence, timely surgery is advocated. But careful follow-up is required as the risk of postoperative complications is also high.

## Figures and Tables

**Table 1:** Patient characteristics (*N* = 13).

Variables	Number	Percentage
*Age*		
Mean	29.3 years	
Range	14–60 years	
*Sex*		
Male	9	69.2
Female	4	30.8
*Laterality of RD in colobomatous eye*		
Right eye	7	53.84
Left eye	4	30.76
Both eyes	1	7.7
*History of prophylactic laser*		
Yes	1	7.7
No	12	92.3
*Associated ocular anomalies*		
Iris coloboma	11	84.61
Microcornea	4	30.76
Nystagmus	3	23.07
Strabismus	3	23.07
Micropthalmus	0	0
Cataract	6	46.15
*Classification of coloboma* ^*∗*^		
Type 1	5	38.46
Type 2	1	7.69
Type 3	7	53.84
Type 4	0	
Type 5	0	
Type 6	0	
Type 7	0	

Grading according to Ida Mann Classification. Abbreviation: *N*: number; RD: retinal detachment; VA: visual acuity. ^*∗*^Ida Mann Classification.

**Table 2:** Surgical record (*N* = 13).

Variables		Number	Percentage
Lensectomy/lens aspiration		6	46.15
Membrane peeling		3	23.07
Identifiable retinal break	Yes	9	69
	No	4	31

Abbreviation: *N*: number.

**Table 3:** Postsurgical record (*N* = 11).

Variables	Number	Percentage
*Complications after surgery*		
Yes	7	63.64
No	4	36.36
*Complications after surgery*		
Recurrent RD	1	7.69
Cataract	1	7.69
Hypotony	0	0
Secondary glaucoma	4	30.7
Band-shaped keratopathy	1	7.69
Phthisis bulbi	0	0

Abbreviation: *N*: number.

## Data Availability

The datasets generated and/or analyzed during the current study are not publicly available because the data are strictly confidential and are the property of institution and the Nepal Health Research Council but are available from the corresponding author on reasonable request.

## Abbreviations

RD: Retinal detachment
RRD: Rhegmatogenous retinal detachment
BCVA: Best-corrected visual acuity
VA: Visual acuity
PVR: Proliferative vitreoretinopathy.
